# Opportunities of mHealth in Preconception Care: Preferences and Experiences of Patients and Health Care Providers and Other Involved Professionals

**DOI:** 10.2196/mhealth.7834

**Published:** 2017-08-17

**Authors:** Matthijs R Van Dijk, Maria PH Koster, Ageeth N Rosman, Regine PM Steegers-Theunissen

**Affiliations:** ^1^ Erasmus MC Obstetrics and Gynecology Rotterdam Netherlands; ^2^ Erasmus MC Pediatrics, Division of Neonatology Rotterdam Netherlands

**Keywords:** Focus group, Lifestyle, mHealth, Nutrition, Personalized medicine, Preconception care

## Abstract

**Background:**

The importance of the preconception period and preconception care (PCC) are broadly acknowledged and the potential benefits regarding health promotion have been studied extensively. PCC provides the opportunity to identify, prevent, and treat modifiable and nonmodifiable risk factors to optimize the health of couples trying to become pregnant. The prevalence of modifiable and nonmodifiable risk factors in these couples is high, but the uptake of PCC remains low.

**Objective:**

The aim of this study is to identify the preferences and experiences of women and men (patients) trying to become pregnant and of health care providers and other involved professionals regarding mobile health (mHealth), in particular the coaching platform Smarter Pregnancy, and its potential role in PCC.

**Methods:**

Patients who participated in the Smarter Pregnancy randomized controlled trial (RCT) and health care providers and professionals also involved in PCC were invited to participate in a qualitative study. The barriers, benefits, and opportunities of big data collection by mHealth were discussed in focus group sessions, prompted with statements regarding PCC.

**Results:**

We composed five focus groups, consisting of 27 patients in total (23 women and 4 men), who participated in the RCT, and nine health care providers and other professionals. Of the patients, 67% (18/27) were familiar with the concept of PCC, but only 15% (4/27) received any form of PCC. A majority of 56% (combined percentages of statements 1 [n=18], 2 [n=11], and 3 [n=16]) of the patients believed in the benefit of receiving PCC, and all agreed that men should be involved in PCC as well. Patients did not have a problem using anonymized data obtained from mHealth tools for scientific purposes. Patients and health care providers and other professionals both acknowledged the lack of awareness regarding the importance of PCC and stated that mHealth provides several opportunities to support clinical PCC.

**Conclusions:**

Our findings substantiate previous studies addressing the low uptake of PCC due to unawareness or lack of perception of its relevance by couples who are trying to become pregnant. The positive judgment and experiences with mHealth, in particular Smarter Pregnancy, will stimulate future research and further development of effective and cost-effective personalized mHealth apps for patients, health care providers, and other professionals as an add-on to clinical PCC.

## Introduction

Since the recommendation of preconceptional folic acid supplement use for the prevention of neural tube defects in the early 1990s, the importance of the preconceptional period in the physiology and pathophysiology of pregnancy outcome and preconception care (PCC) is broadly acknowledged. The potential benefits of health promotion and interventions during this period of at least 14 weeks before conception has been extensively studied [1-3]. PCC can be used to identify, prevent, and treat modifiable and nonmodifiable risk factors and it optimizes the health of couples trying to conceive and, ultimately, the pregnancy outcome [4]. In the Netherlands, PCC is only delivered to a select group of women, mainly those who have a fertility problem or a high risk for adverse pregnancy outcome due to a known genetic or medical condition or a previous poor pregnancy outcome. However, at their own request, couples can receive PCC from a health care professional, but so far only a very small proportion of the general population takes advantage of this. The low uptake of PCC, combined with the high prevalence of unhealthy nutrition and lifestyle behaviors, illustrates the lack of awareness regarding the importance of PCC in couples who are trying to conceive [5-7].

Currently, rapid developments in the field of telemedicine by means of electronic health (eHealth) and mobile health (mHealth) are opening doors to new opportunities to empower patients and health care providers and professionals and to fill the gaps in patient care [8,9]. In 2010, more than 200 million health-related online apps were downloaded, suggesting that mHealth indeed has the potential to reach, inform, and educate a large population [10]. Inherent to such mHealth apps, programs, or services, an enormous amount of data, referred to as “big data,” can be obtained and stored by integrating data of online questionnaires, biofeedback, and diagnostic and monitoring tools. Consequently, big data can be used to study specific populations of interest and is therefore considered to be of great medical and scientific importance in the future [11]. Because nearly all women and men of reproductive age have Internet access and/or own a mobile phone, we believe that mHealth can play a role in providing information that can induce awareness and eventually support the implementation of PCC. Although there are many pregnancy-related mHealth solutions, mHealth solutions focusing on PCC are scarce [12]. Therefore, we consider this study regarding “Smarter Pregnancy” as a pioneer in the field of PCC using mHealth.

The aim of this qualitative study was to explore the preferences and experiences of women and men regarding mHealth, including big data, and its potential role in PCC. Moreover, we discussed these preferences and experiences with health care providers and other involved professionals in the field of PCC.

## Methods

### Participants and Recruitment

All participants (hereafter referred to as “patients” to improve readability) of the Smarter Pregnancy randomized controlled trial (RCT) (ie, fertile and subfertile couples trying to conceive) who completed the first six months of the program or resigned prematurely were invited to participate. The details of the Smarter Pregnancy mHealth platform and the RCT design have previously been published [13,14]. In short, during the Smarter Pregnancy RCT, the intervention group received individual coaching consisting of a baseline screening and a follow-up screening at 6, 12, 18, and 24 weeks regarding nutrition and lifestyle behavior. Coaching also included a maximum of three interventions per week, which consisted of short message service (SMS) text messages and email messages containing tips, recommendations, vouchers, seasonal recipes, and additional questions addressing gender, behavior, first day of last menstrual period, pregnancy status, body mass index, and adequacy of the diet. The control group did not receive the weekly personal coaching after the baseline screening and only received a minimum of feedback on the screening questionnaires at baseline and at 12 and 24 weeks.

For this qualitative study, all potential participants received an email that included an invitation to participate in a focus group session. In this email, we stated that we were interested in their feedback on our mHealth coaching platform and their views on the general concept of mHealth and big data by means of a semistructured interview, prompted with statements about PCC (Table 1).

### Data Collection Procedure

To compose homogeneous focus groups and consequently lower the barrier to participate and increase the response rate, we chose to stratify the groups according to gender, known fertility status, and RCT study group (ie, intervention or control group). We aimed to recruit 6 to 10 patients per group. One week before the meeting, patients received a list of the statements that were going to be discussed during the focus group. At the start of a focus group, patients were asked to fill out a questionnaire regarding their personal information, medical information, and experiences and knowledge on PCC in general.

**Table 1:** Statements used during the focus groups with patients.

Statement	Topic
1. I consider the background information and coaching received by the mHealth program Smarter Pregnancy as useful.	Preconception care
2. Personal coaching by email and text messages is a valuable additive.	mHealth
3. Smarter Pregnancy has a pleasant way of communicating.	mHealth
4. Mobile health is a right method to give preconception care.	mHealth
5. Data obtained from Smarter Pregnancy can be (anonymously) used for other (non)commercial purposes.	Big data

Every focus group meeting took place at the Erasmus MC, University Medical Centre, Rotterdam (the Netherlands), and was preceded by an individual introduction of each patient and a short presentation by a researcher (MRvD) to repeat the aim of the meeting and to ensure confidentiality. During the 2 to 2.5 hour focus group session, a professional moderator (ANR) guided the discussion. The involved researcher (MRvD) took minutes and ensured optimal audio recording.

### Health Care Providers and Professionals

After the focus group sessions with the patients, we also invited health care providers and professionals involved in the fields of reproductive medicine, obstetrics or PCC, policy makers, and representatives of a health care insurance company. Because all focus group sessions with the patients had already been processed and analyzed, health care providers and other professionals were not only asked to discuss their own views regarding PCC, mHealth, and big data, but also to reflect on the patients’ input on these topics.

### Theoretical Framework and Data Analysis

This study is based on a framework described by Fleuren et al [15], which identifies four main stages in innovation processes: dissemination, adoption, implementation, and continuation. These processes can be considered as potential failure points in which the transition from one stage to another is determined by both positive and negative factors (determinants). The framework considers characteristics of the organization, the innovation itself, the end user, and the sociopolitical environment. By using statements prompted during the focus groups, determinants regarding patients’ preferences and experiences were derived. The same was done within the focus group of the professionals; however, specific information from the patient’s focus groups was added and discussed.

All recorded audio was transcribed verbatim, using the minutes as guidance. To perform thematic analysis, a set of preliminary codes was developed from the notes and transcripts and discussed between the researchers involved. Subsequently, the codes were structured to the concepts of determinants as previously described. Two researchers (MRvD and MPHK) coded one transcript independently and then compared the coding to reach consensus. Thereafter, the remaining scripts were coded by MRvD. All coding took place using NVivo version 10 (QSR International, Cambridge, MA, USA).

### Ethical Considerations

All data were anonymously processed. This qualitative study was conducted according to the guidelines laid down in the Declaration of Helsinki and all procedures involving patients were approved by the Medical Ethical and Institutional Review Board of the Erasmus MC, University Medical Centre, Rotterdam, in the Netherlands. Informed consent was obtained from all participants to use anonymized data for analysis.

## Results

### Study Population

A total of 509 patients received an invitation, of which 23 women and 4 men accepted the invitation and were able to participate in four focus groups. Patients who had an indication to receive fertility treatment by means of an in vitro fertilization (IVF) or intracytoplasmic sperm injection (ICSI) were labeled as the IVF-ICSI population, whereas patients who did not receive this treatment were labeled as the general population. Groups were composed as follows:

1. Women, general population (n=5);
2. Women, IVF-ICSI population, intervention group (n=9);
3. Women, IVF-ICSI population, control group (n=9); and
4. Men, IVF-ICSI population (n=4).

Overall, baseline characteristics of these women and men, such as age, ethnicity, educational level, and lifestyle were comparable between patients of the four focus group sessions (Table 2).

The focus group session with health care providers and professionals consisted of nine attendants (ie, a gynecologist, a midwife, a general practitioner, a fertility doctor, a preventive health care center physician, a dietician, a medical advisor from a health insurance company, a representative of the municipality of Rotterdam, and a representative of the Dutch association of parent and patient organizations).

### Preconception Care: Beliefs and Perception

A summary of the patients’ answers on the additional questionnaire at the start of the focus group session is shown in Table 3, illustrating their perceptions and beliefs regarding PCC. Despite the observation that only 67% (18/27) of patients were familiar with the current concept of PCC (ie, a consultation with a health care professional) and only 15% (4/27) received any form of PCC (Table 2), a majority of 56% (combined percentages of statements 1 [n=18], 2 [n=11], and 3 [n=16] in Table 3) indicated the benefits of receiving PCC and adopting a healthy lifestyle when trying to conceive. Whether they believe that if they become pregnant, their child benefits from received PCC remains questionable because only 32% (combined percentages of statements 4 [n=7] and 5 [n=10] of Table 3) agreed with this statement.

**Table 2:** Baseline characteristics of all patients, based on the additional questionnaire (N=27).

Baseline characteristics		General population (n=5)	IVF-intervention group (n=9)	IVF-control group (n=9)	Men (n=4)
Focus group, n		1	2	3	4
Age (years), mean (SD)		33.0 (5.1)	33.7 (5.1)	35.2 (4.3)	43.3 (17.5)
**Marital status, n (%)**					
	Single	—	—	1 (11)	—
	Married or living together, without children	1 (20)	4 (44)	3 (33)	4 (100)
	Married or living together, with children	4 (80)	5 (56)	5 (56)	—
**Ethnicity, n (%)**					
	Dutch or Western	5 (100)	8 (89)	9 (100)	4 (100)
	Non-Dutch, non-Western	—	1 (11)	—	—
**Education, n (%)**					
	None	—	—	—	—
	Low	—	—	1 (11)	—
	Middle	1 (20)	3 (33)	1 (11)	—
	High	4 (80)	6 (67)	7 (78)	4 (100)
Smoking (yes), n (%)		—	—	1 (11)	1 (25)
Alcohol consumption (yes), n (%)		1 (20)	1 (11)	4 (44)	4 (100)
Drug use, n (%)		—	—	—	—
Vitamin use, n (%)		1 (20)	7 (78)	7 (78)	1 (25)
Medication use, n (%)		1 (20)	—	1 (11)	1 (25)
Comorbidity, n (%)		3 (60)	2 (22)	3 (33)	2 (50)
**Mode of conception, n (%)**					
	Spontaneous	3 (60)	1 (11)	1 (11)	—
	Hormonal treatment	—	—	—	—
	IVF or ICSI	—	5 (56)	4 (44)	2 (50)
Nulliparous		1 (20)	6 (67)	5 (56)	4 (100)
Familiar with preconception care, n (%)		2 (40)	8 (89)	4 (44)	4 (100)
Received preconception care, n (%)		2 (40)	—	2 (22)	—

**Table 3:** Patients perceptions and beliefs regarding PCC, prior to the focus group (N=27).

Patients perceptions and beliefs regarding PCC			Focus group, n				Overall, %
			1	2	3	4	
**Preconception care: beliefs and perception**							
	**1. PCC will make me adopt a healthy lifestyle.**						
		Strongly disagree	0	0	0	0	0
		Disagree	0	0	2	1	11
		Neither agree nor disagree	1	1	3	1	22
		Agree	4	8	4	2	67
		Strongly agree	0	0	0	0	0
	**2. Through PCC, I know whether it’s wise to become pregnant.**						
		Strongly disagree	0	0	0	0	0
		Disagree	0	2	2	1	19
		Neither agree nor disagree	1	4	4	2	41
		Agree	4	3	3	0	37
		Strongly agree	0	0	0	1	4
	**3. Through PCC, I’m better prepared to become pregnant.**						
		Strongly disagree	0	0	0	0	0
		Disagree	0	0	1	1	7
		Neither agree nor disagree	1	4	3	1	33
		Agree	4	5	4	2	56
		Strongly agree	0	0	1	0	4
	**4. PCC reduces the risk of complications during pregnancy or labor.**						
		Strongly disagree	0	0	0	0	0
		Disagree	0	1	0	0	4
		Neither agree nor disagree	3	6	5	4	67
		Agree	2	2	3	0	26
		Strongly agree	0	0	0	0	0
	**5. PCC makes my baby more healthy.**						
		Strongly disagree	0	0	0	0	0
		Disagree	0	1	2	0	11
		Neither agree nor disagree	3	6	2	3	52
		Agree	2	2	4	1	33
		Strongly agree	0	0	1	0	4
**Preconception care: logistics**								
	**6. PCC should be obligated**						
		Yes	1	1	1	1	15
		No	4	8	8	3	85
	**7. PCC should be given to:**						
		Women only	0	0	0	0	0
		Men only	0	0	0	0	0
		Women and men	5	9	9	4	100
	**8. When should PCC be reimbursed by an insurance company**						
		Only if a woman has a high-risk (medical) condition	0	5	3	2	37
		Always	5	4	6	2	63
	**9. For whom should PCC be reimbursed**						
		Women only	2	7	4	2	56
		Couples only	0	1	1	1	11
		No opinion	3	1	4	1	33
**Preconception care: conditions and content**							
	**10. Would you prefer anonymous PCC over personal**						
		Yes	0	0	1	0	4
		No	5	9	8	4	96
	**11. PCC should consist of one consultation**						
		Yes	3	2	1	1	26
		No	2	7	7	3	74
	**12. PCC by mobile health can be useful**						
		Yes	4	7	9	4	93
		No	1	1	0	0	7
	**13. PCC can be used unconditionally regarding treatment**						
		Yes	4	5	8	3	74
		No	1	4	1	1	26

### Preconception Care: Logistics

All patients acknowledged that men should be involved in PCC. On the contrary, more than half (15/27, 56%) stated that only the costs of PCC received by women should be reimbursed by the insurance company. Despite the agreement on the importance of PCC, 85% (23/27) stated that it should not be mandatory for couples trying to conceive.

### Preconception Care: Conditions and Content

Most patients (26/27) would not prefer anonymous PCC. Despite previous findings showing a majority stating PCC should not be obligatory, 74% (20/27) stated that PCC should be mandatory as a part of fertility treatment (Table 3).

### mHealth

In general, patients feel comfortable using mobile apps. They believe that using mobile devices in health care is a good development and a modern approach to provide patients with information and background. Most male patients acknowledged that mobile health can be used to substitute for certain parts of regular consultations, especially during fertility treatment, but women emphasized the importance of face-to-face contact and nonverbal communication and stated that mHealth should only be used as an additive to routine clinical care:

It is not necessary to have a face-to-face consultation, if I need to discuss something, I’ll find my way to contact a health care professional.

Man, group 4

If they ask me over the phone through an app, how am I doing, I’ll just say “I’m doing good,” but if they ask me during a consultation, they can see me and notice I’m not doing okay.

Woman, group 1

### Awareness

The most frequently discussed topic during most focus group sessions was “awareness.” Some female patients specifically mentioned the visual feedback, as provided by the Smarter Pregnancy platform, as a trigger and motivator to improve behavior. Knowing they would perform better on the next monitoring questionnaire gave them perceived control, but a high frequency of coaching and incorporated positive feedback is needed to secure adherence to the program and to truly improve awareness:

It makes you more aware of what you’re eating, so when you’re tired you won’t eat an unhealthy snack because you want to reach the best score on the questionnaire.

Woman, group 2

If you truly want to change someone’s behavior, one reminder per week is nice, but not enough to provide sufficient information.

Woman, group 3

Besides all the coaching on what to improve, it’s also nice to hear you’re doing a good job.

Woman, group 2

### Education

Patients agreed that background information on the importance of healthy nutrition and lifestyle as a part of PCC improves awareness, but only if they believe it is trustworthy and preferably evidence-based. The Smarter Pregnancy coaching platform is supported by multiple health care organizations of which the logos are displayed at the website. This was highly appreciated by the patients:

Information found at an online community can be from anyone, and I don’t like to be told what to do by an amateur.

Man, group 4

Using received information as an online reference book, which was always accessible on demand, was suggested to be of great value. Also adding visual content by means of images and videos was considered valuable.

### Personalized Mobile Health

In addition to awareness, participants agreed on the fact that mHealth needs to be highly personalized to be really effective. Impersonal messages or general messages were considered not effective or even countereffective. Men and women both suggested that the psychological aspect of trying to conceive should be integrated in mHealth as well as the functionality of asking online questions to a health care professional:

I guess it would really work if patients can use an accessible “chat function” or “email service” to ask questions to a health care professional.

Woman, group 3

### Big Data

#### Scientific and Commercial Use

All patients were asked their opinion on data obtained through a mHealth platform being used either anonymously or nonanonymously, for scientific purposes. Patients were very willing to support prepregnancy- and pregnancy-related research in general in this way, provided it was anonymous, because they considered it to be helpful for other patients and future parents. Some patients approved of nonanonymous scientific use of their data. Also, “medical-related” companies and organizations, such as hospitals and pharmaceutical companies, were considered to be relevant purposes. On the contrary, most patients did not allow usage of nonanonymous or anonymous data for commercial purposes. There was a general perception that companies or organizations, and health care insurance companies in particular, should not benefit from this data, although one participant mentioned this could be an opportunity to develop profitable PCC:

I am willing to help science, but not willing to help a company sell more of its products.

Woman, group 1

I really value that I decide what to share with the outside world, and with whom.

Woman, group 1

#### Safety and Monitoring

In general, patients were not worried about data leakage due to limited safety of storage by mHealth devices. It was believed that every medical institute itself, or together with governmental support, could guarantee data safety and monitoring.

### Health Care Providers and Professionals

#### Preconception Care

Participating health care providers and professionals also agreed on the general lack of awareness for PCC. To create awareness, the importance of evidence-based information and education was emphasized. For example, consistent online and offline information can educate patients and health care providers and professionals and consequently increase the intrinsic motivation to change certain unhealthy behavior that are not often addressed in health care and PCC. It was suggested that awareness in general can be increased by offering PCC through employers or, even better, through secondary schools integrated within biology or sex education lessons. The health care providers and professionals recognize the fact that patients are familiar with the broad and inconsistent spectrum of online information regarding PCC and notice that especially higher-educated patients use the Internet to obtain information regarding fertility and pregnancy, whereas more lower-educated patients with limited health literacy and the highest health risks prefer to visit a professional first:

It feels like selling something to someone who doesn’t want to buy it. You are trying to convince them of something they don’t believe it’s important.

Gynecologist

With the existence of online communities, patients are “educated” by peers instead of professionals. That is their preconception care.

Fertility doctor

#### mHealth

Health care providers and professionals were familiar with mHealth, especially apps to monitor menstrual cycles, fetal development during pregnancy, and for online questionnaires to identify risk factors for certain conditions (eg, depression and anxiety). In addition to monitoring, they were concerned whether mHealth can reach and educate those who need it the most, for example due to a language barrier. Therefore, it is suggested that developing apps in multiple languages will overcome this. If so, it is believed that mHealth can be used to substitute for certain elements of routine consultations (eg, nutrition and lifestyle recommendations). Replacing consultations by alternative techniques, such as video chat, is believed to be an upcoming development, but is currently not appreciated due to the lack of technical support and security.

All health care providers and professionals were very enthusiastic about the concept of using an online questionnaire, such as the one incorporated in the Smarter Pregnancy platform, including a link between the given answers and the patient’s medical record. The prospect of having all the results before a face-to-face PCC consultation was considered very useful and timesaving. Moreover, providing questionnaires to patients was in itself already thought to increase awareness:

Monitoring over time is very useful, because knowing whether a patient is improving gives the opportunity to give them a compliment, which can be very helpful.

Gynecologist

#### Big Data

The health care providers and professionals unanimously agreed that big data can be of great medical and scientific importance. By obtaining detailed information on target groups and populations, interventions can be designed and clinical care can be tailored at specific behaviors, needs, or risk factors of specific patient groups. Although the health care providers and professionals were aware of the perception of patients toward the use of big data, they believed that commercial use could also be beneficial in creating large-scale awareness.

## Discussion

### Principal Findings

This qualitative study addressed the preferences and experiences of patients and health care providers and other professionals regarding PCC in general and mHealth in particular. Based on the four focus group sessions with patients the following can be summarized:

Patients are familiar with PCC in general and confirm that there is a lack of awareness regarding the importance.

Patients believe that mHealth can play a role in PCC, especially regarding awareness and providing evidence-based information, but mainly as an additive to standard care with face-to-face contact with a health care professional.

Patients also believe that mHealth should be personalized, customized, and tailored to their needs, risks, and behaviors to reach its full potential and become truly effective.

Patients approve that data obtained from mHealth, referred to as big data, can be used anonymously for scientific purposes.

The health care providers and other professionals agree on the potential role of mHealth in PCC, especially as an effective tool to inform and educate couples to improve awareness of the importance of PCC care in general. They are optimistic about the concept of mHealth integrated into the patients’ medical records, but emphasize that the current situation is not suitable for this innovation due to the lack of technical support.

### Comparison With Literature

Our findings correspond with existing literature, in which low uptake of PCC due to unawareness or a lack of perception of relevancy by couples trying to conceive have been described [16-18]. Concerning mHealth and its role in PCC, previous studies have suggested that tailored interventions may improve the uptake of PCC, especially when added to standard care [19,20]. Currently, the development and uptake of commercial and non-evidence-based apps continues, whereas there is an ongoing debate on the efficacy of mHealth in general, because the scientific merit is questionable due to the absence of robust evidence [13,20-23]. Therefore, many studies are conducted to provide scientific evidence on the effectiveness of mHealth interventions in general [14,24-28]. To our knowledge, the perception of patients regarding the use of big data for scientific purposes has not been described before.

Regarding the preferences and experiences of patients using mHealth interventions in general, our findings are in line with previous studies. The personalized character and credibility of mHealth interventions have recently been described to be important to enhance adherence to therapy and nutrition and lifestyle recommendations [29-31].

### Strengths and Limitations

Patient involvement during the designing phase of an intervention is essential, followed by end user participation and evaluation of an intervention to further improve customization [32,33]. Consequently, the main strength of this study is the involvement of several end users of our mHealth platform (ie, participants of the Smarter Pregnancy RCT), including the participation of men. Also, we included multiple health care providers and other professionals, representing various organizations and professions in the field of PCC, which is an important strength. These professionals were able to state their own opinion, substantiated by the policy of the organization or profession they represent. Due to the careful stratification and composition of the focus groups, we created a safe environment for the patients in which the structured discussion took place. Furthermore, with the presence of a professional moderator, we were able to give all participants the opportunity to express and interactively discuss their opinions, experiences, and feelings equally and without any consequences.

The most important limitation to address is the low acceptance rate resulting in a very small number of patients in total and per focus group, although this can be considered as confirmation of the main underlying problem: the lack of knowledge and awareness regarding PCC. This, together with the involvement of end users, may also introduce selection bias; the patients involved in this study are generally highly educated and probably more engaged with the topic because they already participated in a previous study regarding mHealth and PCC. Although this bias is hard to overcome when conducting qualitative studies in general, and especially in this field of research with a population of interest that is very hard to reach, it needs to be addressed because it could influence the external validity of the results.

### Conclusions

Overall, we conclude that patients and health care providers and professionals believe that mHealth has several unique opportunities for PCC. Our findings imply that future research should focus on the development of mHealth apps as an add-on to standard care, preferably integrated or connected to patients’ medical records, allowing health care providers and other professionals to become involved and support their patients. The first step to increase awareness would be to provide evidence-based information, followed by providing apps or programs containing this information, but also tailored to individual conditions. Therefore, patient involvement and end user participation will be indispensable in designing effective interventions.

## Abbreviations

eHealth: electronic health
ICSI: intracytoplasmic sperm injection
IVF: in vitro fertilization
mHealth: mobile health
PCC: preconception care
RCT: randomized controlled trial
